# Prostatic Acid Phosphatase (PAP) Antibodies to Treat Castration-Resistant Prostate Cancer

**DOI:** 10.3390/ijms27146133

**Published:** 2026-07-09

**Authors:** Alexander Kirschenbaum, Pamela Cheung, Shen Yao, J. Andrew Duty, Thomas Kraus, Thomas Moran, Alice C. Levine

**Affiliations:** 1 Department of Urology, Icahn School of Medicine at Mount Sinai, New York, NY 10029, USA; 2 Division of Endocrinology, Diabetes and Bone Diseases, Department of Medicine, Icahn School of Medicine at Mount Sinai, New York, NY 10029, USA; 3 Laboratory of Therapeutic Antibody Development, Drug Discovery Institute, Icahn School of Medicine at Mount Sinai, New York, NY 10029, USA; 4 Department of Microbiology, Icahn School of Medicine at Mount Sinai, New York, NY 10029, USA

**Keywords:** prostate cancer, transmembrane prostatic acid phosphatase, progenitor/stem cell, metastatic, castration-resistant, hypoxia, tumor microenvironment, 5′ectonucleotidase, adenosine, adenosine receptors

## Abstract

Prostate cancer (PCa) is the most common cancer and the second leading cause of cancer death in American men. Most patients with metastatic disease respond initially to androgen deprivation therapy (ADT) but almost inevitably progress to castration-resistant prostate cancer (CRPC). Identification of markers and drivers of Metastatic CRPC (mCRPC) that (a) represent a progenitor-type cancer cell population, (b) persist in castration-resistant disease, (c) are actionable targets expressed on the cell surface, and (d) are induced by hypoxia is required to facilitate the development of novel targeted therapies. We identified prostatic acid phosphatase (PAP), particularly the transmembrane form (TMPAP), as one such potential target. PAP is both a phosphatase and a 5′ectonucleotidase that generates adenosine. PAP is a human tumor marker first described in 1936 and is still used as an important prognostic marker for advanced metastatic prostate cancer. Our group recently reported that the transmembrane form of the protein (TMPAP) is expressed in CRPC and can serve as a potential therapeutic target. We identified a lead human anti-TMPAP antibody clone 3D8 (3D8-Ab). 3D8-ADCs (Antibody Drug Conjugates) and 3D8-Ab were tested for their ability to reduce tumor size/volume in a xenograft model. The human PAP-expressing PCa cell line VCaP, originally derived from a vertebral metastasis from a patient with CRPC, was inoculated subcutaneously into SCID mice. Treatment with either 3D8-Ab or 3D8-ADC significantly reduced tumor size and increased animal survival. These data indicate that targeting PAP with monoclonal antibodies either alone or conjugated to toxins has the potential to treat CRPC.

## 1. Introduction

Prostate cancer (PCa) is the second most common cancer among men in the United States and the second leading cause of cancer-related death in men, with an estimated 35,000 deaths annually in the U.S. [1]. For localized, clinically significant PCa, there are many treatment options, including focal therapy, surgery, and radiotherapy, with five-year survival approaching 100%. However, patients with locally advanced, high-grade PCa, treated with surgery or radiotherapy in conjunction with androgen deprivation therapy (ADT), have a high rate of progression leading to increased cancer-specific mortality. Patients with metastatic PCa, either hormone-naive or castration-resistant, have the poorest prognosis, and new therapeutic approaches are needed [2,3].

PAP is a protein phosphatase and 5′ectonucleotidase expressed in normal and cancerous prostate epithelial cells that has served as a diagnostic marker for prostate cancer [4]. PAP expression persists in most mCRPC bone metastases and may play a causal role in castrate-resistant osteoblastic metastasis and mineralization, possibly via effects on the RANK/RANKL/OPG system [5,6,7,8,9]. More recent evidence demonstrates that in addition to secretory PAP, a membrane-bound splice variant, transmembrane PAP (TMPAP), is detected in mouse and human prostates [10]. The two forms of PAP are quite similar in structure, and both have phosphatase and 5′nucleotidase activity. However, secretory PAP lacks the hydrophobic transmembrane domain. TMPAP incorporates an alternative terminal sequence, which includes a hydrophobic transmembrane anchor (22 amino acids) that tethers the protein to the membrane, followed by a short cytosolic C-terminal [11]. Mechanistically, the ectonucleotidase function of both isoforms generates extracellular adenosine, which promotes cancer cell growth, migration, and invasion [12]. Adenosine signaling via the adenosine receptor 2B (A2BR) and adenosine receptor 3 (A3R) promotes tumor progression, angiogenesis, metastasis, and escape from immune attack in diverse cancers, including prostate cancer [13,14,15,16,17,18]. Processes defining the hypoxic landscape of the tumor microenvironment (TME) increase adenosine 1000-fold. Studies utilizing a human glioblastoma cell line demonstrated that (1) extracellular adenosine production was higher under hypoxia than normoxia, mainly due to (2) hypoxia-inducible factor-2 alpha (HIF-2a) induction of PAP expression, driving adenosine production from adenosine monophosphate (AMP) and resulting in (3) maintenance of the cancer stem cell phenotype and enhanced cell migration, invasion and EMT [19,20,21,22,23].

The prostate gland develops from epithelial buds arising from the urogenital sinus (urethra) immediately below the bladder [24]. Epstein et al. reported on a series of bladder cancers and tumors with mixed glandular and transitional features that expressed PAP, even those derived from female patients [25]. This data indicates that neither androgens nor androgen receptor (AR) signaling are needed for PAP expression and implies that PAP-expressing cells represent a more progenitor/stem cell-like phenotype. Elevated levels of PAP protein have been reported to be an independent predictor of tumor recurrence, suggesting that PAP-expressing cells represent an aggressive PCa progenitor population [26].

Castration-resistant PCa (CRPC) is heterogeneous. The main castration-resistant PCa cells are cancer progenitor/stem cells that tend to reside in the hypoxic niche. Under the pressure of androgen ablation and chemotherapy, this population undergoes selection and plasticity, leading to the lethal phenotype. CRPC-resistant cells fall into five categories based on androgen receptor (AR) and neuroendocrine (NE) markers: (1) AR+/NE−; (2) AR low/NE−; (3) AR+/NE+; (4) AR−/NE−; and (5) AR−/NE+. The first 4/5 of these resistant phenotypes express PAP; the pure NE phenotype (AR−/NE+) is PAP negative.

To expand the therapeutic armamentarium available to men with advanced PCa, additional therapeutic targets that persist in castration-resistant disease and drive growth in the hypoxic TME must be identified. We demonstrated that TMPAP is one such target [27] and have developed monoclonal antibodies to TMPAP for therapeutic use. We herein demonstrate the in vivo efficacy of TMPAP antibodies and antibody conjugates on the reduction in tumor growth and increased survival in a xenograft model of CRPC.

## 2. Results

### 2.1. Production of Monoclonal Antibodies Against PAP

**Figure 1:**
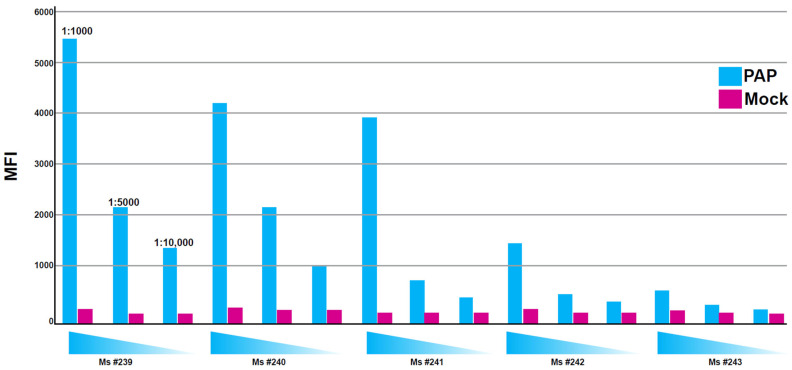
Serum screening. Mice were immunized and boosted with soluble PAP and bled. Sera were diluted and tested for binding on PAP-transfected Expi293 cells and on mock-transfected cells.

A group of five VelocImmune^®^ mice was immunized with commercially sourced, soluble PAP (Cell Sciences, cat # 14633B). This PAP is purified from human seminal fluid and is >98% pure by SDS page. The immunizations were done with an adjuvant. After two boosts, the mice were bled, and the antibody titer was measured by flow cytometry on Expi293 cells transfected with PAP (Figure 1). Mouse number 239 was fusion 1, and mouse number 240 was fusion 2.

Individual clones of mAb were selected using the Hamilton ClonaCell EasyPick STAR Robotic System, and those specific for PAP were identified by flow cytometry on Expi293 cells transfected with a plasmid encoding human PAP. ELISA was also performed as a primary screen. Positive clones in the primary screen were verified in a secondary screen before they were expanded and further evaluated.

### 2.2. Characterization of Monoclonal Antibodies

**Figure 2:**
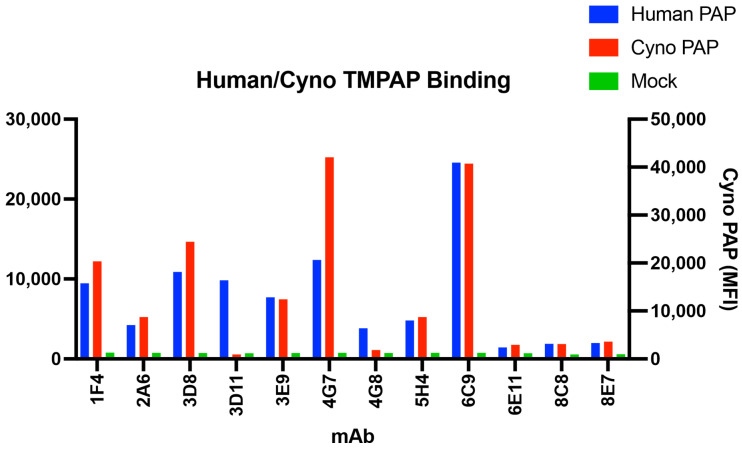
Human/Cyno TMPAP Binding Expi293 cells were transfected with transmembrane PAP from humans or cynomolgus monkeys. Positive clones binding to both forms of PAP were chosen for further characterization.

A total of 53 positive hybridomas from fusions 1 and 2 were captured by ELISA and flow cytometry. Of these positive clones, 42 were bound by flow cytometry. Antibodies were rescreened via flow cytometry on VCaP cells, cynomolgus monkey PAP (cynoPAP)-transfected Expi293 cells, and mock-transfected cells. To further select clones for cross-reactive activity, twelve clones were selected for binding to both human and cynoPAP (Figure 2). These antibodies were then isotyped and sequenced to assess variable gene usage and clonality (identical CDR3 and VDJ gene usage). We were able to determine that clones 6E11, 8C8, and 8E7 were identical. All others were unique, leaving a set of eight lead clones for characterization. 

We then ranked the binding affinity of the antibodies to the soluble dimer and the monomeric transmembrane isoform. While the ultimate intent was not to find single reactive antibodies, if we found an antibody that did not bind to the soluble PAP, its value would increase. However, the affinities of the antibodies were all similar, except for 2A6, which was not a top performer in cell binding. We then ranked the affinity by cell binding. Expi293 cells transfected with PAP were stained with descending doses of the PAP antibodies for 30 min, washed and stained with goat anti-mouse IgG (heavy only)-APC-secondary antibody before washing and undergoing flow cytometry. Table 1 shows that the antibodies did have different EC50s for binding to cells and magnitudes of binding to various isoforms.

### 2.3. Antibody Drug Conjugation

We selected a lead clone, 3D8, that had affinity for both sPAP and TMPAP, and produced it recombinantly on an IgG4 isotype. We then conjugated two well-known auristatin toxins to it through standard cysteine chemistry; both have been used in past ADC candidates in clinical trials for prostate cancer. We also conjugated an isotype control (IgG4). These were linked via well-known, complementary linkers for each payload (NJBio, Inc., Princeton, NJ, USA). Four conjugated species were generated, which included 3D8-vedotin (mc-vc-PABC-MMAE), 3D8-mafodotin (mc-MMAF) and a conjugated isotype control for each. Drug antibody ratios (DARs) ranged from 3 to 3.9, and percent monomers ranged from 98 to 99%. No free drugs were detected.

### 2.4. In Vitro Cytotoxicity of ADCs

**Figure 3:**
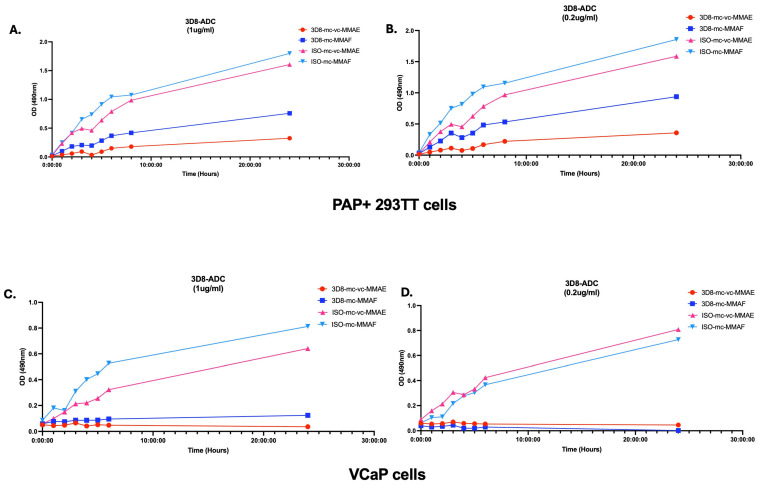
Human/Cyno TMPAP Binding Either 293TT or VCaP cells (1 × 10^4^) were incubated with 1 μg/mL (**A**,**C**) or 0.2 μg/mL (**B**,**D**) for 72 h. Time elapsed after adding MTS substrate and a representative sample of duplicates are shown.

To test cytotoxicity, an MTS assay was used to determine the number of live cells after incubation with 3D8-ADC conjugates or isotype control conjugates for 72 h (Figure 3). MTS tetrazolium and its formazan product, at an absorbance of 490 nm, is directly proportional to the number of living cells. VCaP or PAP-transfected adherent 293TTcells (1 × 10^4^ cells) both showed significant reduction in absorbance at 0.2 μg/mL (Figure 3A,C) or 1ug/mL (Figure 3B,D). After the incubation with ADCs and addition of MTS substrate, absorbance was measured every hour up to 6 h and then once again at 24 h. 3D8-ADC-treated cells maintained their cytotoxicity for up to 24 h, even though a small rebound was seen in the PAP-transfected 293TT population. This possibly could have been due to the expansion of untransfected cells.

**Figure 4:**
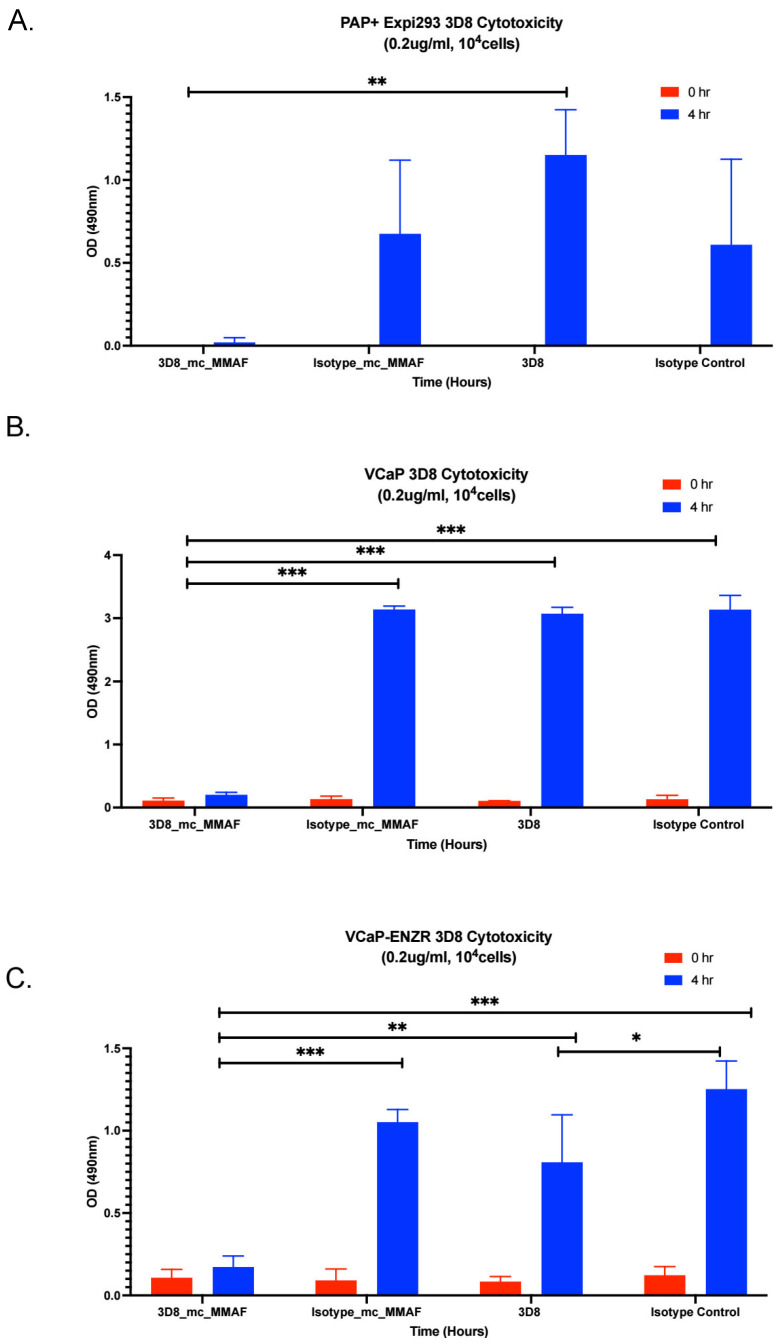
Cytotoxic effects of 3D8-mcMMAF in PAP+ Expi293, VCaP, and VCaP-ENZR cells. (**A**–**C**) PAP+ Expi293, VCaP, and VCaP-ENZR cells were treated with 3D8-mcMMAF, isotype-mcMMAF, unconjugated 3D8, or isotype control at 0.2 µg/mL (10^4^ cells/well), and cell viability was assessed by MTS assay at 0 and 4 h by measuring OD490. Data are presented as mean ± SEM. Statistical significance was determined at the 4 h time point using ordinary one-way ANOVA followed by Tukey’s multiple comparisons test. * *p* < 0.05, ** *p* < 0.01, *** *p* < 0.001.

Further, unconjugated 3D8 (3D8-Ab) (0.2 μg/mL) was assessed for cytotoxicity compared to 3D8-mafodotin (3D8-mc-MMAF) and was compared to TMPAP-transfected Expi293 cells or endogenously expressed VCaP cells. 3D8 alone did not show any in vitro cell cytotoxicity compared to the 3D8-mcMMAF. 3D8-Ab alone was also assessed for cytotoxicity in enzalutamide-resistant VCaP subline (VCaP-Enz) cells and found not to be as cytotoxic as 3D8-mcMMAF (*p* < 0.01), which also displayed significant cytotoxicity when compared to both isotope controls (*p* < 0.001). Unconjugated 3D8 alone did demonstrate, though, a modest but statistically significant increase in cytotoxicity relative to isotype control in VCaP-Enz cells (*p* < 0.05) (Figure 4).

### 2.5. In Vivo Efficacy and Toxicity

**Figure 5:**
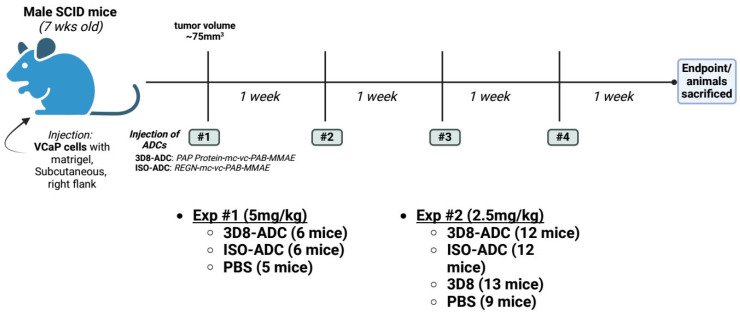
In vivo experimental timeline.

To assess the therapeutic effects of reducing tumor volume for 3D8-ADCs and 3D8-Ab, we carried out experiments at 5 and 2.5 mg/kg doses of ADC or antibody alone (see timeline in Figure 5). The first experiment at the higher dose, 5mg/kg, was performed with three arms (3D8-ADC, isotype-ADC, and PBS). In the second experiment, the dose was lowered to 2.5mg/kg and a fourth arm was added to receive treatment with 3D8-Ab alone. While both payload-toxins showed equally significant in vitro cytotoxicity, Vedotin was chosen as the payload-toxin due to a more favorable cleavable linker.

**Figure 6:**
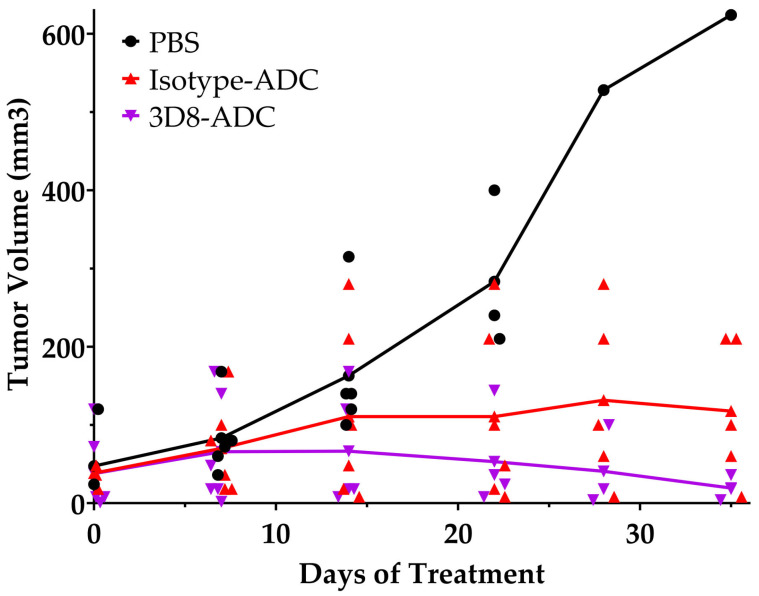
In vivo mouse experiments at high dose (5 mg/kg): 3D8-ADC vs. PBS and isotype-ADC controls. Mice were treated with 5 mg/kg of antibody, i.p. once a week for four weeks after the tumor size reached ~75 mm^3^ for the majority of mice. Tumor size was assessed by calipers in 3 dimensions each week. PBS n = 5; isotype-ADC n = 6; 3D8-ADC n = 6.

In the first experiment, 3D8-ADC and isotype-ADC were compared to PBS control for efficacy. There was tumor size reduction seen in both the isotype-ADC and 3D8-ADC arms, but there were not enough surviving control animals at the end of the experiment (only 1 of 5 survived) to carry out statistical analysis. In addition, the doses of 3D8-ADC and isotype-ADC were concluded to be toxic, given the 50% survival rate in these arms at the end of the experiment. This may be due to the toxicity of the drug conjugate (vedotin), indicating non-specific off-target effects and/or the toxicity related to free drug release from the ADC complex. The increased mortality in the high-dose group is unlikely to be due to higher tumor burden, as the animals with lower tumor volumes at the start of the injections were the ones that did not survive. In addition, the two animals in the 3D8-ADC arm that started the injection cycle with larger tumors > 100 mm^3^ not only survived but also saw the most significant tumor reduction compared to any of the arms. The PBS-treated arm showed the most significant tumor growth once tumors passed the 75mm^3^ threshold (Figure 6). This is also correlated with the lowest survival rate.

In the second experiment, lower doses of Ab and ADC were utilized, and an extra treatment group was introduced, 3D8-Ab (unconjugated to vedotin toxin). Age-matched male SCID mice were injected with 10^6^ VCaP cells subcutaneously on the right flank, and treatments were initiated once tumors were palpable. Mice were divided into 4 treatment arms: (1) PBS, (2) Isotype-ADC, (3) 3D8-Ab and (4) 3D8-ADC; at 2.5 mg/kg i.p., once per week for 4 weeks, and tumor size was assessed in three dimensions. Mice were further divided into two groups based on the size of the tumor at initiation of treatment.

**Figure 7:**
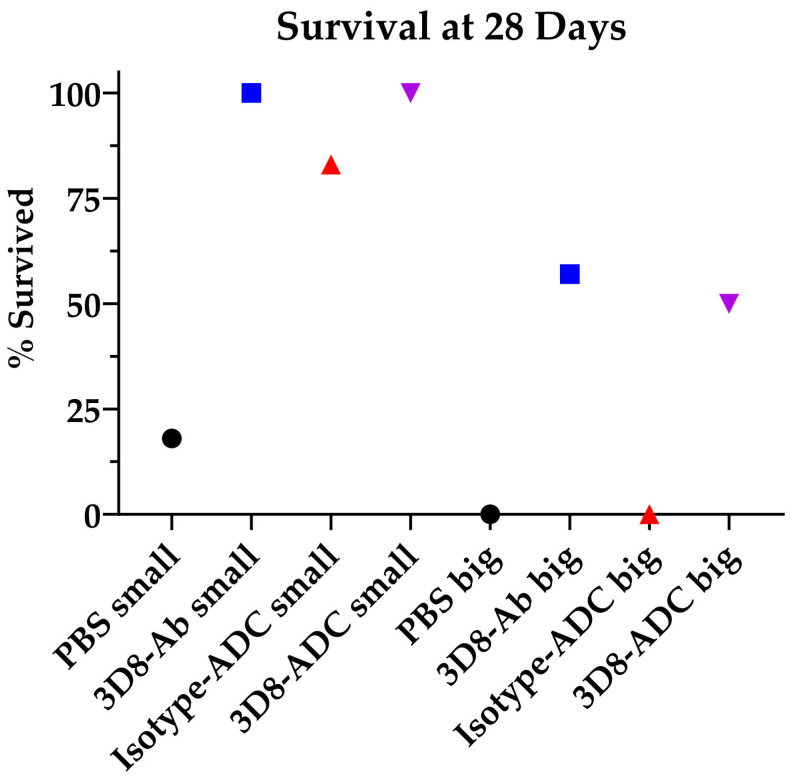
Survival percentages at 28 days at lower dose (2.5 mg/kg): 3D8-Ab or 3D8-ADC vs. PBS and isotype-ADC controls.

**Figure 8:**
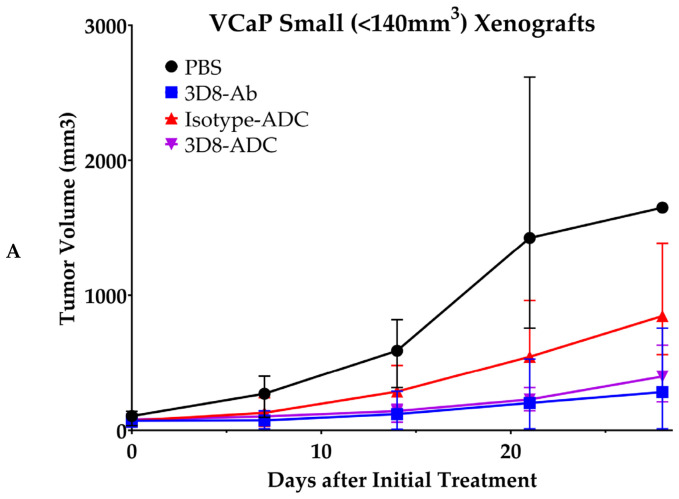

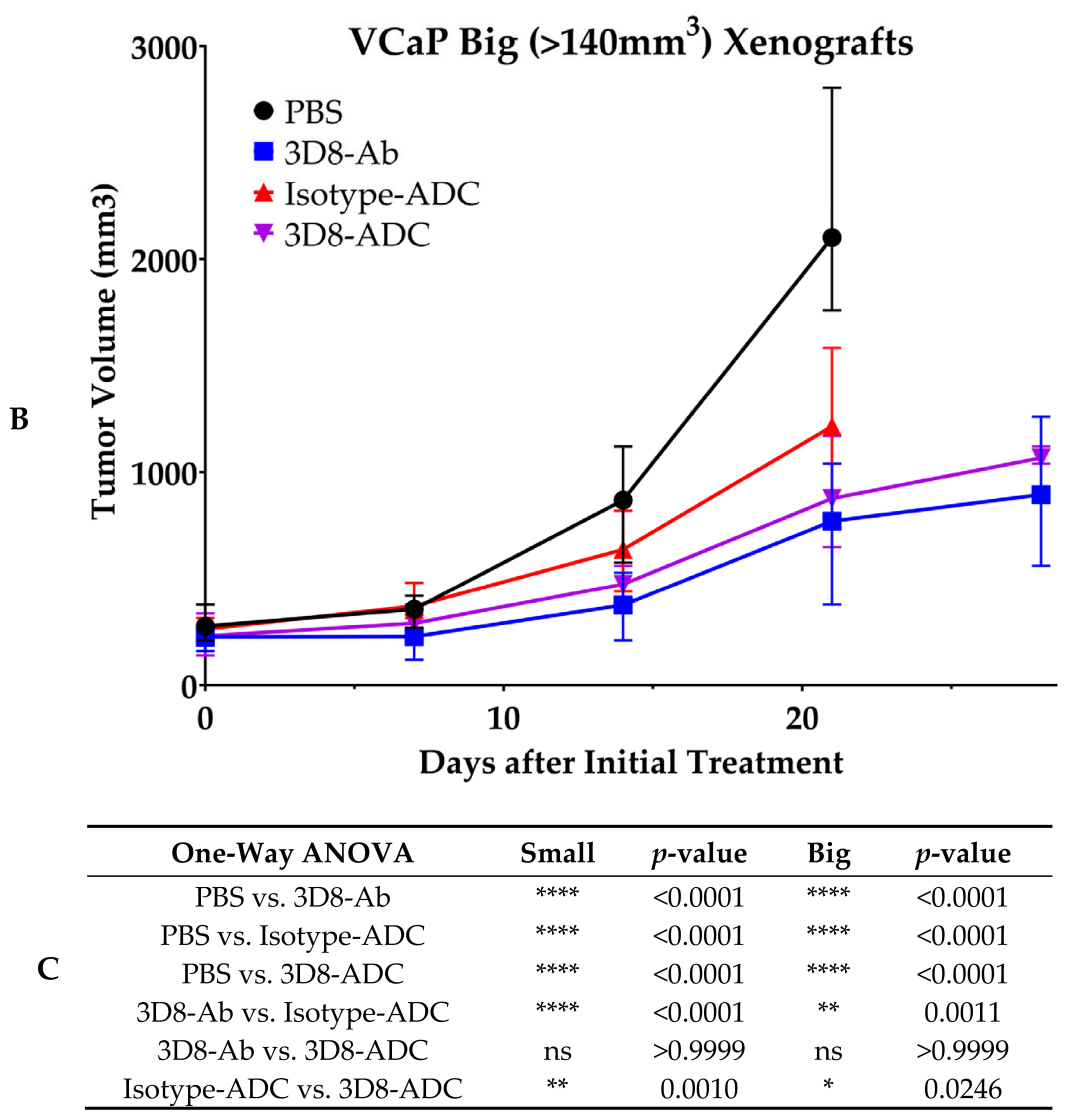
In vivo mouse experiments at lower dose (2.5 mg/kg): 3D8-Ab or 3D8-ADC vs. PBS and isotype-ADC controls. 3D8-Ab and 3D8-ADC improved survival of mice with small and large tumors. Lowering the dose to 2.5 mg/kg reduced toxicity and showed significantly reduced tumor volumes for all antibody treatment groups, including 3D8-Ab (without toxin). Mice were further divided into 2 groups based upon size of tumor at initiation of treatment: (**A**) big (>140 mm^3^), PBS n = 4; 3D8-Ab n = 5; isotype-ADC n = 5; 3D8-ADC n = 6; (**B**) small (<140 mm^3^), PBS n = 5; 3D8-Ab n = 8; isotype-ADC n = 7; 3D8-ADC n = 6. (**C**) One-way ANOVA with multiple comparisons was performed, and *p*-value was assessed by Graphpad Prism 9.0 software. Asterisks indicate significant differences; *p*-values also shown to indicate degree of differences between groups.

We observed that the lower dose resulted in reduced toxicity and increased survival. The survival of the animals treated with 3D8-Ab and 3D8-ADC was higher compared to the PBS or ADC groups (Figure 7). The isotype-ADC reduced tumor growth compared to PBS, possibly indicating off-target effects associated with this dose of vedotin toxin. The most significant tumor reduction was observed in all antibody treatment groups, including 3D8-Ab and 3D8-ADC, compared to PBS and isotype-ADC (Figure 8). The effectiveness of 3D8-Ab was unexpected as our in vitro studies did not show the efficacy of the antibody alone. However, in vivo, 3D8-Ab appeared as effective as 3D8-ADC. The differences in tumor growth suppression for both 3D8-Ab and 3D8-ADC vs. controls are even more significant in smaller tumors, but are clearly statistically significant in all tumors regardless of size.

**Figure 9:**
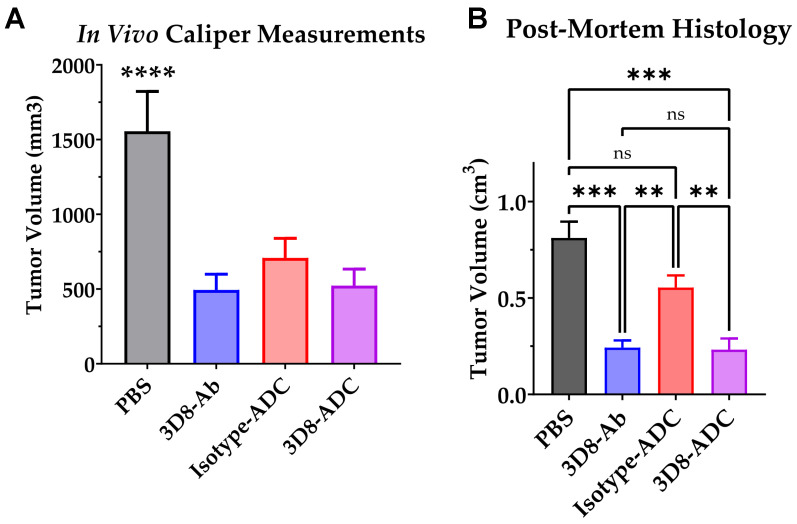

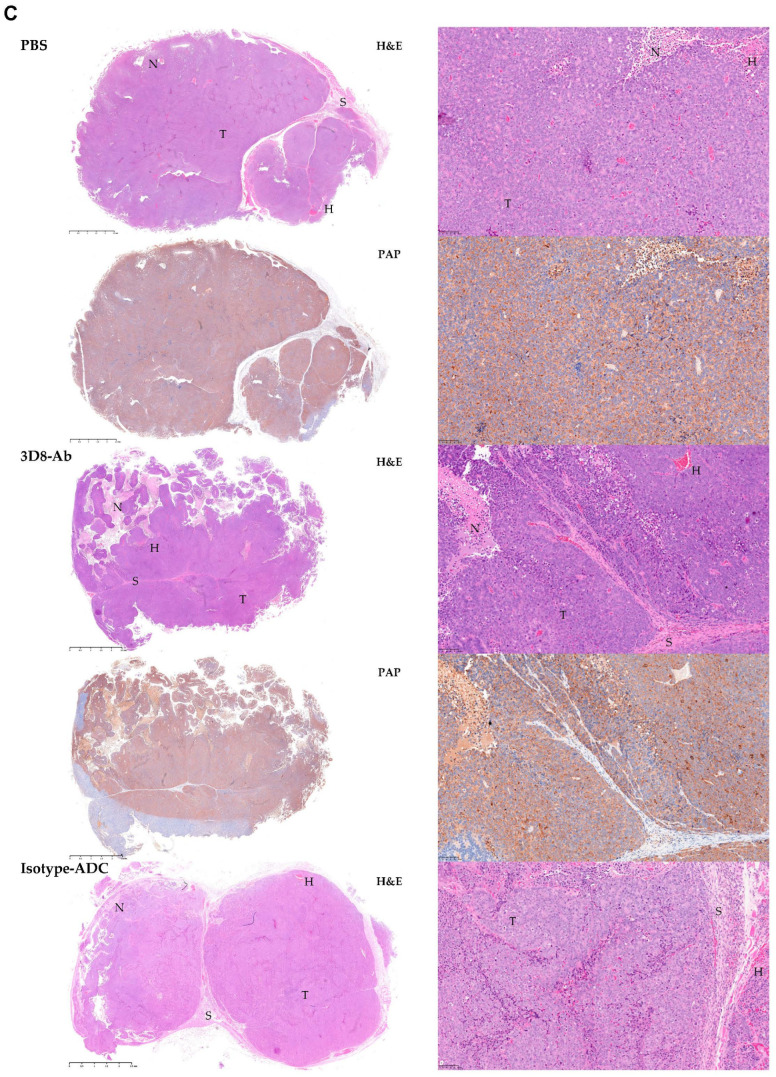

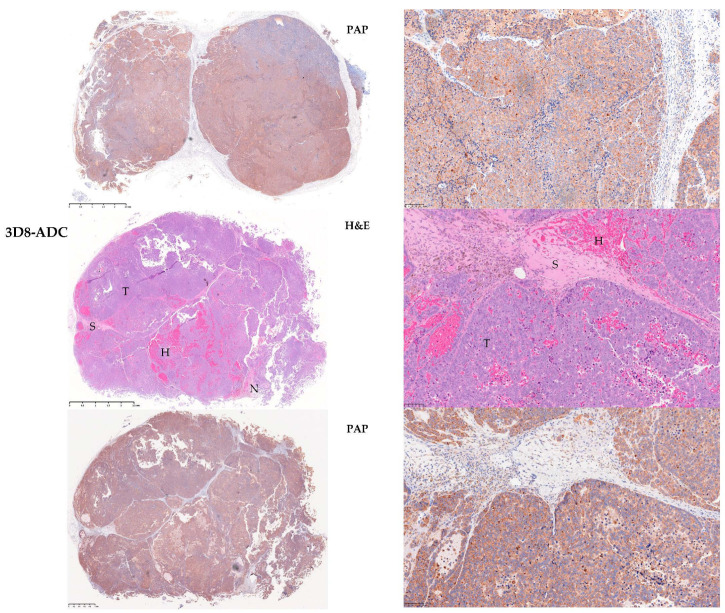
Tumor volume as assessed with calipers vs. post-mortem histology. (**A**) Tumor volume assessed by calipers in vivo at 3 weeks of treatment. (**B**) Tumor volume (mm^3^) as assessed by histology of sectioned VCaP tumors post-mortem. One-way ANOVA with multiple comparisons was performed, with *p*-values assessed by Graphpad Prism 9 software. ** *p* < 0.01, *** *p* < 0.001, **** *p* < 0.0001 (**C**) Representative samples of post-mortem histology, demonstrating more hemorrhage and necrosis in 3D8-Ab- and 3D8-ADC-treated xenografts on H&E, and continued PAP expression in all 4 groups. T = Tumor; H = Hemorrhage; S = Mouse Stroma; N = Necrosis. Scale bar = 2.5mm (left) and 0.25mm (right).

Tumor size was initially assessed with calipers in vivo (Figure 9A), and there was a statistically significant difference between PBS and the other groups. Post-mortem tumors were analyzed histologically (Figure 9B), and the amount of tumor vs. necrosis was quantified. In this analysis, the reduction in actual tumor volume with 3D8-Ab and 3D8-ADC vs. the two control groups (PBS and isotype-ADC) was statistically significant, as some of the volume in the 3D8-Ab- and 3D8-ADC-treated tumors, as measured by calipers, included hemorrhagic and necrotic tissue. Figure 9C demonstrates representative samples of each group. Hemorrhage (H) and necrosis (N) are more abundant in the 3D8-Ab and 3D8-ADC specimens, as seen in both H&E. Tumors from all four groups expressed PAP, even in the necrotic areas.

**Figure 10:**
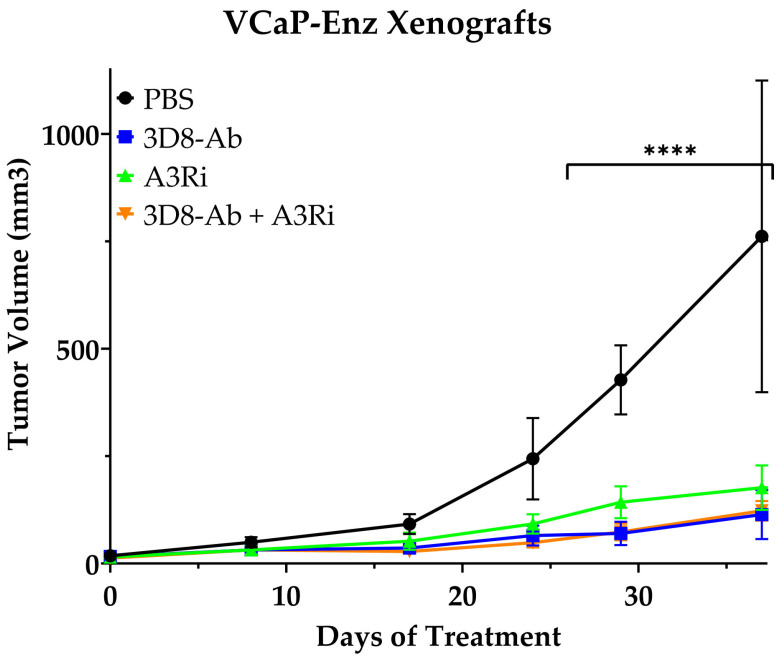
Comparison of 3D8-Ab and A3 adenosine receptor inhibitor (A3Ri) alone or in combination on VCaP-Enz tumor growth in vivo. PBS n = 4; 3D8-Ab n = 4; A3Ri n = 4; 3D8-Ab + A3Ri n = 4 **** *p* < 0.0001.

Our group previously reported that the enzalutamide-resistant subline of VCaP, VCaP-Enz, expresses higher levels of TMPAP than the parental line. This cell line more closely mimics CRPC in that it is resistant to anti-androgen treatment. TMPAP has been shown to increase tumor growth and invasiveness via generation of adenosine in glioblastoma cells [21]. Accordingly, we compared the effectiveness of 3D8-Ab vs. an adenosine receptor inhibitor (A3Ri) on VCaP-Enz growth in vivo. We demonstrated that both treatments, 3D8-Ab and A3Ri, significantly reduced tumor growth compared to PBS control. Combination therapy with 3D8-Ab plus A3Ri did not demonstrate additive anti-tumor effects beyond that seen with each of the therapies alone. However, 3D8-Ab significantly reduced tumor volume in the same manner as A3Ri, implying that some of the growth inhibition observed with 3D8-Ab may be due to inhibition of the 5′ectonucleotidase activity of sPAP, resulting in lower adenosine levels (Figure 10).

## 3. Discussion

While traditional prostate cancer treatments continue to be refined, overall survival rates have not improved significantly over the past decade. Identification of markers and drivers of PCa lethal phenotypes that (a) represent a progenitor-type cancer cell population, (b) persist in castration-resistant disease, (c) are actionable targets expressed on the cell surface, and (d) are induced by hypoxia in the TME is required to facilitate the development of novel targeted therapies to overcome resistance and lethal disease. We identified prostatic acid phosphatase (PAP) as one such potential target [27]. We herein demonstrate the effectiveness of unique monoclonal antibodies to PAP, both conjugated and unconjugated to ADCs, for the treatment of CRPC in a xenograft model.

Out of 53 human monoclonal antibodies generated against human PAP, we discovered a unique lead clone, 3D8, which had a strong affinity for both forms of PAP (sPAP and TMPAP) and also showed internalization after binding. This clone was generated recombinantly and used as a candidate monoclonal antibody for a linker–payload antibody conjugation to deliver auristatin toxins to PAP-expressing tumors.

The VCaP cell line, originally derived from a vertebral metastasis from a patient with CRPC, is an ideal model to study the therapeutic effects of our antibody as it has high PAP expression and contains 4/5 of CRPC-resistant phenotypes: (1) AR+/NE−; (2) AR low/NE−; (3) AR+/NE+; and (4) AR−/NE−. PAP is highly expressed in all four CRPC phenotypes of human pathologic specimens and is notably upregulated by hypoxia [26]. Antibody-conjugates demonstrated cytotoxicity in vitro, when tested on both the VCaP and VCaP-enzalutamide resistant human PCa cell lines. Unconjugated 3D8-Ab did not demonstrate cytotoxicity in vitro. However, our in vivo studies showed that our 3D8-Ab and 3D8-ADC significantly inhibited growth in both cell lines in SCID mice. 3D8-Ab appeared to be as effective as the 3D8-ADC in vivo, implying that it was inhibiting some critical activity of PAP itself involving interactions with the tumor microenvironment (TME), which was not detectable with the cell lines in vitro. We hypothesize that the in vivo efficacy of the 3D8-Ab is due to inhibition of the 5′ ectonucleotidase activity of PAP, resulting in lower adenosine generation in hypoxia. This is supported by the data demonstrating that 3D8-Ab alone was as effective as an inhibitor of adenosine receptor 3 (A3Ri) in terms of in vivo tumor growth reduction utilizing the VCaP-Enz cell line. Ongoing experiments will determine whether this mechanism underlies the efficacy of our antibodies in the hypoxic tumor microenvironment.

Limitations of our study include the use of only two cell lines and the lack of a metastatic model. However, since the VCaP model mimics many of the features seen in mCRPC in men, including heterogeneous resistant phenotypes and a tendency to produce osteoblastic bone metastases, we anticipate that these antibodies may have broad therapeutic applicability. Moreover, PAP expression is highly prostate-specific; therefore, off-target toxicity should be low.

Antibodies to one PCa target, prostate-specific membrane antigen (PSMA), have been developed [28]. This work culminated in an FDA-approved radioligand therapy for prostate cancer, Lutetium-177 vipivotide tetraxetan PSMA therapy (Pluvicto), a new theragnostic medicine for advanced metastatic prostate cancer [29]. Our novel TMPAP antibodies may have even broader therapeutic potential than PSMA, as PAP is expressed in normal, differentiated, and resistant PCa cells. Therapeutic PAP-Ab-ADCs may be efficacious in both hormone-naive and castration-resistant tumors in locally advanced or metastatic disease, as PAP is strongly expressed in progenitor cells associated with high-grade PCa. Early application of this therapy may eradicate the progenitor population and thus prevent the emergence of the neuroendocrine phenotype.

## 4. Materials and Methods

### 4.1. Production and Characterization of Monoclonal Antibodies Against PAP

A group of 5 VelocImmune^®^ mice was immunized with commercially sourced, soluble PAP (Cell Sciences cat # 14633B, Newburyport, MA, USA). This PAP is purified from human seminal fluid and is >98% pure by SDS page. The immunizations were done with an adjuvant. After two boosts, the mice were bled, and the antibody titer was measured by flow cytometry on Expi293 cells transfected with PAP. Individual clones of monoclonal antibodies (mAbs) were selected using the Hamilton (Reno, NV, USA) ClonaCell EasyPick STAR Robotic System, and those specific for PAP were identified by flow cytometry on Expi293 transfected with a plasmid encoding human PAP. Positive hybridomas were captured by ELISA and Flow cytometry as a primary screen, and positive clones were rescreened by flow and isotyped, screened on VCaP cells, cynoPAP, and mock-transfected cells before they were expanded and further evaluated for clonality.

### 4.2. Antibody Sequence Analysis of Heavy Chain Variable Region and Junctional Diversity

Sequencing of variable heavy and kappa chains was obtained using SMARTer 5′ RACE technology (Takara Bio, San Jose, CA, USA) adapted for immunoglobulins to amplify the variable genes from the heavy and kappa chains. Briefly, RNA was extracted from each hybridoma using an RNeasy Mini Kit (Qiagen #74004, Venlo, Netherlands), followed by first-strand cDNA synthesis using constant gene-specific 3′ primers (GSP1) based on the specific mouse isotype of the hybridoma and incubation with the SMARTer II A Oligonucleotide and SMARTscribe reverse transcriptase (Takara Bio, 634858). [GSP1 Primers (5′-3′): mG1-AGAGGTCAGACTGCAGGACA, mG2a-CTTGTCCACTTTGGTGCTGC, mG2b-GACAGTCACTGAGCTGCTCA, mG2b-GACAGTCACTGAGCTGCTCA, mcK-CCAACTGTTCAGGACGCCAT]. PCR amplification of the first-strand cDNA product was then performed using SeqAmp DNA Polymerase (Takara, 638504) with a nested 3′ primer (GSP2 Primer) to the constant genes and a 5′ universal primer (kit provided) based on universal primer sites added to the 5′ end during cDNA generation. [GSP2 Primers (5′-3′): mG1-CCCAGGGTCACCATGGAGTT, mG2a-GGTCACTGGCTCAGGGAAAT, mG2b-CTTGACCAGGCATCCCAGAG, mG3-GACAGGGCTCCATAGTTCCATT, mCk-CTGAGGCACCTCCAGATGTTAAC]. Purified PCR products were submitted for Sanger sequencing using 3′ constant gene primers (GeneWiz, South Plainfield, NJ, USA). Sequence results were BLASTed against the IMGT human databank of germline genes using V-Quest (http://imgt.org/).

### 4.3. Isotyping and Monoclonal Antibody Purification

Isotyping for the constant gene of the antibodies was performed with the Mouse Immunoglobulin Isotyping Kit (BD#550026 Franklin Lakes, NJ, USA) as per the manufacturer’s protocol. Monoclonal antibodies were purified by FPLC on an ÄKTA pure FPLC system on protein G affinity columns (HiTrap-1 mL, GE/Cytiva, #17-0404-01, Marlborough, MA, USA). They were dialyzed against PBS and quantified by both BCA and OD at 280 nm. Recombinant preparation and purification, including cloning variable segments into mammalian expression vectors containing human IgG4/human kappa constant regions, of lead antibody 3D8 and isotype control were performed by Regeneron Pharmaceuticals (Tarrytown, NY, USA).

### 4.4. Quantitation of Monoclonal Antibodies

Monoclonal antibodies were quantified using label-free biolayer interferometry (BLI) on an Octet Red96 (ForteBio, Sartorius Fremont, CA, USA). Briefly, optically capable biosensors conjugated with either protein A or G were dipped into supernatants containing mAbs. Supernatants were measured undiluted and diluted 1:10 in conditioned media and compared to an isotype standard, diluted in conditioned media in a 1:2 dilution series ranging from 100 μg/mL to 1.56 μg/mL. Standard curves were analyzed in ForteBio Data Analysis Software v.10 (ForteBio) using a 5-parameter logistic (5PL) dose–response curve fitting model on the initial binding slopes. mAb concentrations were then calculated from the standard curve. Diluted samples were compared to undiluted samples after application of the respective dilution factors. Total concentrations were averaged together from the diluted and undiluted samples.

### 4.5. In Vitro Cytotoxicity Assay

CellTiter 96 AQueous One Solution Cell Proliferation MTS Assay (Promega, Madison, WI, USA) was used to measure cell toxicity in PAP-expressing cells under the manufacturer’s provided protocol. The kit measures MTS tetrazolium and its formazan product, at absorbance of 490nm, which is directly proportional to the number of living cells. VCaP or PAP-transfected adherent 293TT cells (1 × 10^4^ cells) were plated in a 96-well plate and treated with ADCs for 72 h at a concentration range of 0.2 or 1ug/mL which was chosen by independent experiments to be optimal. After the incubation with ADCs, MTS substrate was added, and absorbance was measured every hour up to 6 h and then once again at 24 h. Background absorbance (MTS substrate alone) was subtracted.

### 4.6. Immunohistochemistry

Immunohistochemical reagents were obtained from Vector Laboratories (Burlingame, CA, USA). Formalin-fixed tissues were embedded in paraffin. Staining was carried out using the avidin–biotin complex method as previously described [26].

### 4.7. Xenograft Tumor Model

VCaP (ATCC# CRL-2876, Manassas, VA, USA) and VCap-EnzR (Signma-Aldrich SCC421, St. Louis, MO, USA) were maintained in DMEM with 10% fetal calf serum at 37 °C in 5% CO_2_, as per recommendation. 1 × 10^6^ VCaP cells were collected and resuspended in half the volume of Matrigel. A total of 100 uL of this cell suspension was injected into the flank of 7-week-old NOD/SCID mice (6–12 per arm) (Charles River, Wilmington, MA, USA). Treatments were initiated once tumors became palpable, followed by tumor measurements with calipers and mouse body weight twice weekly. The treatment groups were as follows: (1) Control (PBS), (2) Isotype-ADC, (3) 3D8-ADC, (4) 3D8-Ab at 5 mg/kg (experiment 1) or 2.5 mg/kg (experiment 2), all given intraperitoneally once weekly, for at least 4 weeks. The adenosine receptor inhibitor treatment groups were as follows: (1) Control (PBS injection), (2) the A3R antagonist MRS1220 (Cat # 1217, Tocris Biosciences, Bristol, UK) at 0.05 mg/kg, intraperitoneally two times weekly, (3) 3D8-Ab at 2.5 mg/kg intraperitoneally once weekly, and (4) MRS1220 at 0.05 mg/kg two times weekly and 3D8-Ab at 2.5 mg/kg once weekly, all given intraperitoneally for 4 weeks. Mice were euthanized at the end of the experiment (or if tumor volume exceeded 1 cm in diameter).

### 4.8. Statistical Methods

Data analysis and visualization were performed using Graphpad Prism 9.0. Differences among groups were analyzed using ordinary one-way ANOVA followed by Tukey’s multiple comparisons test, significance as indicated by * *p* < 0.05, ** *p* < 0.01, *** *p* < 0.001, **** *p* < 0.0001.

## 5. Patents

U.S. provisional patent application number 64/016,935; ANTI-TMPAP ANTIBODIES; filed 25 March 2026.

## Figures and Tables

**Table 1:** Antibody affinity for sPAP/TMPAP.

mAb	Affinity (K_D_) sPAP (M)	Affinity (EC_50_) TMPAP (M)
1F4	5.31 × 10^−10^	3.84 × 10^−9^
2A6	6.59 × 10^17^	5.94 × 10^−9^
3D8	2.04 × 10^−10^	5.18 × 10^−10^
3E9	1.32 × 10^−10^	6.36 × 10^−9^
4G7	2.89 × 10^−10^	1.53 × 10^−9^
5H4	5.53 × 10^−9^	1.49 × 10^−9^
6C9	1.17 × 10^−9^	2.65 × 10^−8^
8C8	3.67 × 10^−10^	4.07 × 10^−8^

## Data Availability

The original contributions presented in this study are included in the article. Further inquiries can be directed to the corresponding author.

## Abbreviations

The following abbreviations are used in this manuscript:

PAP	Prostatic Acid Phosphatase
Ab	Antibody
ADC	Antibody Drug Conjugate
VCaP	Vertebral Cancer of the Prostate
mAbs	Monoclonal Antibodies
